# The Effects of Soft-Segment Molecular Weight on the Structure and Properties of Poly(trimethylene terephthalate)-block-poly(tetramethylene glycol) Copolymers

**DOI:** 10.3390/polym17202781

**Published:** 2025-10-17

**Authors:** Hailiang Dong, Yuchuang Tian, Junyu Li, Jiyou Shi, Jun Kuang, Wenle Zhou, Ye Chen

**Affiliations:** 1 State Key Laboratory of Advanced Fiber Materials, College of Materials Science and Engineering, Donghua University, Shanghai 201620, China; lddonghailiang@163.com; 2 Sinopec Shanghai Research Institute of Petrochemical Technology Co., Ltd., Shanghai 201208, China; tianych.sshy@sinopec.com (Y.T.); shijy.sshy@sinopec.com (J.S.); zhouwl.sshy@sinopec.com (W.Z.)

**Keywords:** direct esterification, soft segment, block copolymers, molecular sequence structure

## Abstract

A series of PTT-*b*-PTMG copolyesters was synthesized via direct esterification followed by melt polycondensation using purified terephthalic acid (PTA), bio-based 1,3-propanediol (PDO), and poly(tetramethylene glycol) (PTMG) of varying molecular weights (650–3000 g/mol). The resulting materials were comprehensively characterized in terms of chemical structure, molecular weight, thermal behavior, phase morphology, crystalline architecture, and mechanical performance using a range of analytical techniques: Fourier-transform infrared spectroscopy (FTIR), ^1^H-NMR, gel permeation chromatography (GPC), differential scanning calorimetry (DSC), thermogravimetric analysis (TGA), wide-angle X-ray scattering (WAXS), small-angle X-ray scattering (SAXS), dynamic mechanical thermal analysis (DMA), tensile testing, and other standard physical methods. FTIR, ^1^H-NMR, and GPC data confirmed the successful incorporation of both PTT-hard and PTMG-soft segments into the copolymer backbone. As the PTMG molecular weight increased, the average sequence length of the PTT-hard segments (L_n,T_) also increased, leading to higher melting (T_m_) and crystallization (T_c_) temperatures, albeit with a slight reduction in overall crystallinity. DMA results indicated enhanced microphase separation between hard and soft domains with increasing PTMG molecular weight. WAXS and SAXS analyses further revealed that the crystalline structure and long-range ordering were strongly dependent on the copolymer composition and block architecture. Mechanical testing showed that tensile strength at break remained relatively constant across the series, while Young’s modulus increased significantly with higher PTMG molecular weight—concurrently accompanied by a decrease in elongation at break. Furthermore, the elastic deformability and recovery behavior of PTT-*b*-PTMG block copolymers were evaluated through cyclic tensile testing. TGA confirmed that all copolyesters exhibited excellent thermal stability. This study demonstrates that the physical and mechanical properties of bio-based PTT-*b*-PTMG elastomers can be effectively tailored by adjusting the molecular weight of the PTMG-soft segment, offering valuable insights for the rational design of sustainable thermoplastic elastomers with tunable performance.

## 1. Introduction

Thermoplastic poly(ether-ester) elastomers (TPEEs) represent a distinctive class of thermoplastic polymers that combine the processing advantages of thermoplastics with the elastic performance of rubbers [1,2]. Their molecular architecture comprises two chemically distinct blocks: a low-glass-transition-temperature (T_g_) amorphous soft segment and a high-melting, highly crystalline hard segment [3,4]. The thermodynamic incompatibility between these segments drives microphase separation, causing the hard domains to aggregate into physically crosslinked, thermally reversible networks [5,6]. This dual-phase morphology endows TPEEs with exceptional mechanical strength, resilience, and thermal stability, rendering them ideal for demanding applications where performance under harsh conditions is critical [2]. Owing to their balanced properties—including excellent low-temperature flexibility, high stiffness, and robust mechanical behavior—TPEEs have become key engineering materials. They are extensively employed across diverse sectors such as automotive components, transportation systems, sporting equipment, battery components, and biomedical devices [2,7,8,9,10,11].

Thermoplastic poly(ether-ester) elastomers (TPEEs) were first described as block co-polymers in 1954 [12] and later commercialized in 1972 under the trade name Hytrel^®^ [13]. Hytrel^®^ is a well-known example, typically composed of crystalline poly(butylene terephthalate) (PBT) as the hard segment and poly(tetramethylene glycol) (PTMG) as the soft segment [14]; besides, other aromatic polyesters—such as poly(ethylene terephthalate) (PET) [15], poly(butylene isophthalate) (PBI) [16], and poly(butylene 2,6-naphthalene dicarboxylate) [17]—have also been employed as hard segments in TPEE formulations. Notably, these aromatic components are predominantly derived from non-renewable fossil feedstocks [18].

Driven by growing environmental awareness, concerns over fossil fuel depletion, and the demand for sustainable materials, research into bio-based polymers has expanded rapidly in recent years [13,19,20]. Among these, bio-based 1,3-propanediol (Bio-PDO)—commercially produced by DuPont via microbial fermentation of renewable starch, has emerged as a key building block [21]. Poly(trimethylene terephthalate) (PTT) named Sorona by DuPont which is polymerized through the polycondensation of PDO and terephthalic acid. Notably, PTT contains 37% renewable carbon content, and its production consumes 30% less energy and generates 63% fewer greenhouse gas emissions per pound compared to nylon 6 [22]. The presence of three methylene units in its repeat unit imparts greater chain flexibility to PTT, resulting in superior properties—such as enhanced tensile elastic recovery—relative to PBT and PET [23,24]. Consequently, PTT has attracted significant interest as a bio-based hard segment in thermoplastic polyester elastomers (TPEEs). For instance, DuPont’s Hytrel^®^ RS incorporates PTT as the rigid phase and uses Cerenol™—a polyether derived from Bio-PDO—as the soft segment [25]. Pioneering work by Szymczyk and colleagues [10,23,25,26,27,28], has extensively explored the synthesis and structure–property relationships of PTT-based multiblock copoly(ether-ester)s, employing either poly(ethylene glycol) (PEG) [26] or poly(tetramethylene glycol) segments [29] as the flexible component. Their studies also examined the effect of PTMG molecular weight (e.g., 1000 and 2000 g/mol) on material performance [25]. Additionally, Yang et al. investigated how composition influences the crystallization behavior of PTT-b-PEOT copolymers [30]. Despite these advances, a comprehensive and systematic investigation into the impact of PTMG’s soft-segment molecular weight—particularly across a broad range—on the phase morphology, thermal behavior, and mechanical properties of poly(trimethylene terephthalate)-block-poly(tetramethylene oxide) (PTT-*b*-PTMG) co-polymers remains notably scarce.

In this study, poly(ether-ester) multiblock copolymers were synthesized using poly(trimethylene terephthalate) (PTT) as the rigid segment and poly(tetramethylene glycol) (PTMG) as the soft segment. The effect of varying the molecular weight of the PTMG-soft block—specifically at 650, 1000, 2000, and 3000 g/mol—on the structure and properties of the resulting PTT-*b*-PTMG segmented copolymers was systematically investigated. A comprehensive suite of characterization techniques was employed, including Fourier-transform infrared spectroscopy (FTIR), ^1^H-nuclear magnetic resonance (NMR), gel permeation chromatography (GPC), differential scanning calorimetry (DSC), thermogravimetric analysis (TGA), dynamic mechanical thermal analysis (DMA), wide-angle X-ray scattering (WAXS), small-angle X-ray scattering (SAXS), and mechanical testing.

## 2. Materials and Methods

### 2.1. Materials

Purified terephthalatic acid (PTA) was obtained from Zhejiang Yisheng Petrochemicals Co., Ltd. (Ningbo, China). 1,3-propanediol (PDO) was purchased from Guangdong Qingda Zhixing Biotechnology Co., Ltd. (Guangzhou, China). Poly(tetramethylene oxide) glycol with molecular weight of 650, 1000, 2000 and 3000 g/mol were purchased from Shanghai Linghan Scientific Instrument Co., Ltd. (Shanghai, China). Ti catalyst was obtained from Sinopec Shanghai Research Institute of Petrochemical Technology Co., Ltd. (Shanghai, China). All the other reagents were purchased from Sinopharm Chemical Reagent Co., Ltd. (Shanghai, China).

### 2.2. Synthesis of PTT-b-PTMG Copolymers

The synthesis was carried out in two sequential stages: esterification followed by melt polycondensation. In the first stage, terephthalic acid (TPA, 431.6 g, 2.6 mol) and 1,3-propanediol (1,3-PDO, 316.2 g, 4.16 mol) were charged into a 2.5 L stainless steel reactor under a nitrogen atmosphere. The esterification reaction was performed at 220–250 °C for 3–4 h under N_2_ and a pressure of 0.25 MPa. Once the amount of water collected as a byproduct exceeded 90% of the theoretical yield—indicating near-complete formation of bis(3-hydroxypropyl) terephthalate—PTMG (400 g), antioxidant Irganox 1010 (0.05 wt% relative to the melt), and titanium-based catalyst STI-T1 (0.35 wt% relative to the melt) were introduced into the reaction mixture. The temperature was then gradually raised to 230 °C and held for 45 min to complete the transesterification step. Following this, excess 1,3-PDO was removed by distillation as the temperature was further increased and the system pressure was progressively reduced.

The second stage—melt polycondensation—was conducted at 260 °C under high vacuum (60 Pa). Throughout this phase, the melt viscosity was monitored via torque measurements, and the reaction was terminated once a consistent viscosity plateau was reached at 260 °C. The resulting polymer was extruded from the reactor under a nitrogen purge. Additional PTT-*b*-PTMG copolymers were prepared using the identical protocol, with only the PTMG molecular weight varied. The series denoted as PTT-*b*-PTMG(650), PTT-*b*-PTMG(1000), PTT-*b*-PTMG(2000) and PTT-*b*-PTMG(3000) contain PTMG of 650, 1000, 2000 and 3000 g/mol, respectively.

### 2.3. Characterizations

The chemical structures of the synthesized copolymers were confirmed by ^1^H-NMR spectroscopy, performed on a 500 MHz Bruker spectrometer (Bruker Corporation, Karlsruhe, Germany) at 25 °C, using trifluoroacetic acid-d (CF_3_COOD) as the solvent.

FTIR analysis of the PTT-*b*-PTMG copolymers was conducted using a Bruker Tensor 27 spectrometer (Bruker Optik GmbH, Karlsruhe, Germany) with attenuated total reflectance (ATR) mode. Spectra were collected over the wavenumber range of 4000–400 cm^−1^ at a resolution of 1 cm^−1^, with 64 scans averaged per sample

Intrinsic viscosity [η] of the samples was determined at 25 °C using an Ubbelohde viscometer in a phenol/1,1,2,2-tetrachloroethane (60:40 *w*/*w*) solvent mixture. The polymer concentration was 0.5 g/dL.

Molecular weight distributions were assessed by gel permeation chromatography (GPC) in 1,1,1,3,3,3-hexafluoro-2-propanol (HFIP) as the eluent. The system comprised a Waters 1515 isocratic HPLC pump, Waters 2707 autosampler and Waters 2414 refractive index detector (operated at 35 °C) (Waters Corporation, Milford, MA, USA). Separation was achieved using a PSS PFG guard column followed by two PFG Linear-XL columns (7 μm, 8 × 300 mm) in series, maintained at 40 °C (Waters Corporation, Milford, MA, USA). The flow rate was 1 mL/min. Calibration was performed using nine narrow-dispersity poly(methyl methacrylate) standards (Polymer Laboratories, Coventry, UK).

Thermal stability was evaluated via thermogravimetric analysis (TGA) on a TA Instruments Q500 (TA Instruments, New Castle, DE, USA) apparatus under a nitrogen atmosphere, heating from 40 to 700 °C at 10 °C/min.

Thermal transitions and crystalinity were analyzed by differential scanning calorimetry (DSC) using a TA Q200 instrument (TA Instruments, New Castle, DE, USA). Samples underwent a three-step thermal protocol: initial heating to 250 °C (10 °C/min), isothermal hold for 3 min, cooling to room temperature (10 °C/min), and a second heating to 250 °C at the same rate. Melting and crystallization enthalpies were calculated from the integrated areas of the endothermic and exothermic peaks, respectively, and normalized per gram of sample.

Wide-angle X-ray scattering (WAXS) measurements were performed on a Xeuss 3.0 system (Xenocs SAS, Grenoble, France) equipped with a Cu Kα source (λ = 0.154 nm) and Ni filter. Diffraction patterns were collected at room temperature with sample-to-detector distances optimized for the wide-angle regime.

Small-angle X-ray scattering (SAXS) data were collected on the same Xeuss 3.0 instrument with adjustable sample-to-detector distances (42.5–4600 mm) to cover the desired q-range. The X-ray wavelength and filtering were identical to WAXS. Scattering images were corrected for dark current and transmission, followed by azimuthal integration to obtain 1D I(q) curve. The long period (L), representing the average distance between adjacent phase (correlation between same block phase), was calculated using the Bragg relation L = 2π/q, where q is the position of the primary scattering peak in the Lorentz-corrected SAXS profile.

Dynamic mechanical thermal analysis (DMA) was carried out using a Polymer Laboratories MK II instrument (Malvern Panalytical, Shropshire, UK) in three-point bending mode. The tests were conducted at a frequency of 1 Hz, with the temperature ramped from −100 °C to 170 °C at a rate of 3 °C/min.

The tensile properties were evaluated in accordance with DIN 53544 using dumbbell-shaped specimens prepared by compression molding. All tests were performed at room temperature with a constant crosshead speed of 50 mm/min, and each reported value is the mean of seven independent measurements. Cyclic tensile tests were carried out on an Instron 3366 universal testing machine (Instron, Norwood, MA, USA) fitted with a 5 kN load cell, a non-contact optical long-travel extensometer (Zwick, Ulm, Germany), and the Bluehill 2 software (Instron, Norwood, MA, USA) was used. To assess elastic deformability and recovery behavior, specimens underwent repeated loading–unloading cycles following a protocol adapted from a previously published method [25].

## 3. Results and Discussion

### 3.1. Structure and Composition of PTT-b-PTMG Copolymers

**Figure 1:**
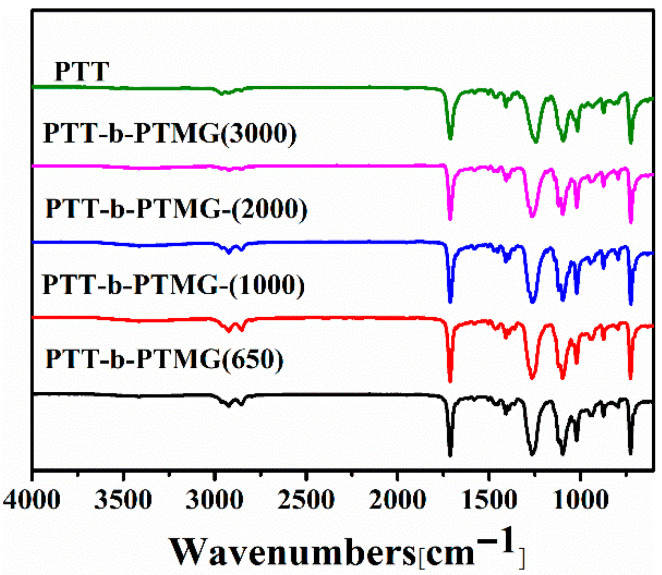
Infrared spectrum of PTT-*b*-PTMG.

Figure 1 displays the FT-IR spectra of PTT-*b*-PTMG copolymer and homo-PTT. As shown in Figure 1, the infrared spectra of the PTT-*b*-PTMG sample and PTT exhibit similar absorption peaks. The absorption peak at 2942 cm^−1^ corresponds to the stretching vibration of CH_2_, while the strong absorption peak at 1710 cm^−1^ is attributed to the vibrational absorption of the carbonyl group (C=O). The peak at 1270 cm^−1^ represents the skeletal vibration of the ester group (=C−O) [31], and the absorption peak at 1100 cm^−1^ corresponds to the stretching vibration of C−O in the ester group, confirming the presence of ester groups. The strong peak at 724 cm^−1^ is associated with the wagging vibration absorption of CH_2_ in aromatic polyester. The peaks at 1020 cm^−1^ and 876 cm^−1^ correspond to the in-plane and out-of-plane deformation vibrations of C−H in the 1,4-substituted benzene ring, respectively. Additionally, the FT-IR spectrum of the PTT-*b*-PTMG copolymer exhibits a new absorption peak at 2854 cm^−1^, which is characteristic of the methylene group (−CH_2_−) in the polyether segment [11]. Moreover, the PTT-*b*-PTMG copolymer sample shows a significantly larger peak area at 1100 cm^−1^ compared to PTT, indicating a higher content of ether bonds (C−O−C) in the copolymer.

**Figure 2:**
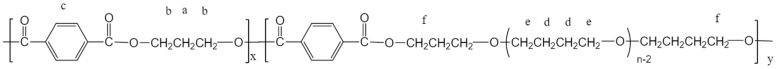
Chemical structural formula of PTT-*b*-PTMG.

**Figure 3:**
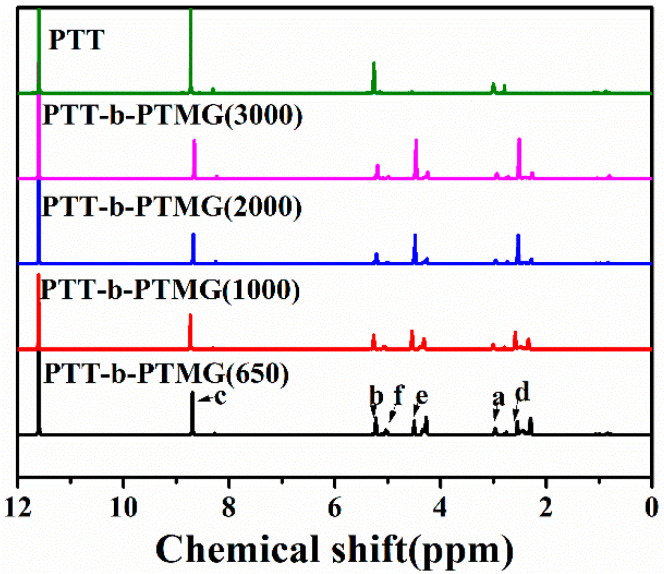
^1^H-NMR spectra of PTT-*b*-PTMG.

The chemical structure PTT-*b*-PTMG is shown in Figure 2 and Figure 3. The signal at 8.6 ppm (peak c, 4H) is assigned to aromatic protons. Peaks at 2.9 ppm (peak a, 2H, –C–CH_2_–C–) and 5.2 ppm (peak b, 4H, –O–CH_2_–C–) originate from the methylene protons of the short-chain diol (1,3-propanediol, PDO). Signals at 4.4 ppm (peak e, –O–CH_2_–C–) and 2.5 ppm (peak d, –C–CH_2_–C–) are attributed to methylene protons within the PTMG-soft segments. A minor resonance at 5.0 ppm (peak f, –COO–CH_2_–C–) arises from methylene protons adjacent to ester linkages in the PTMG block. The peak at 11.5 ppm corresponds to the residual proton of trifluoroacetic acid-d (d-TFA), used as the solvent.

**Figure 4:**
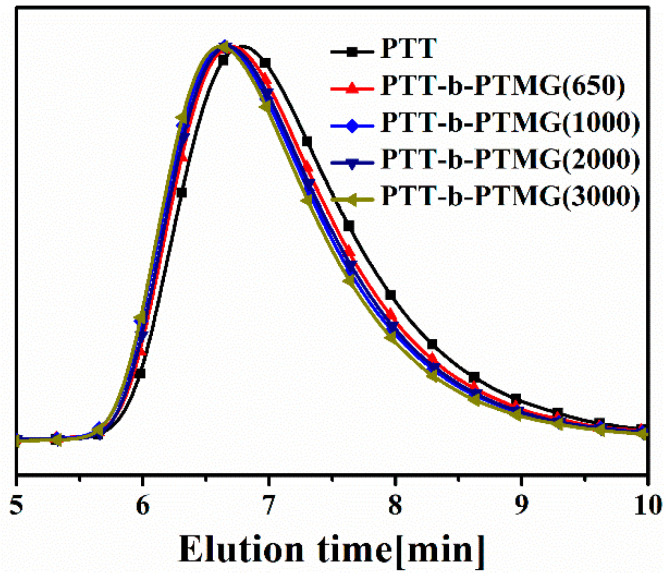
GPC of PTT-*b*-PTMG.

The composition and physical properties of the synthesized PTT-*b*-PTMG copolymers are summarized in Table 1. The intrinsic viscosity [η] of these copolyesters ranged from 1.22 to 1.25 dL/g—significantly higher than that of homopolymer PTT (0.92 dL/g) prepared under identical conditions. This increase suggests that the incorporation of PTMG segments leads to copolymers with molecular weights comparable to or higher than that of PTT homopolymer. This inference is supported by GPC results as shown in Figure 4 and Table 1: the number-average molecular weights (*M*_n_) of the copolymers varied between 38,600 and 43,600 g/mol, with polydispersity indices (PDI) in the narrow range of 1.86–1.90, indicating a moderate increase in molecular weight with the incorporation of PTMG.

The molar percentage of the hard segment (PTT) (*M*_h_), the molar percentage of the soft segment (PTMG), and the weight ratio of the soft segment (*W*_s_) were calculated using Equations (1)–(3), where the average segment length of the hard segment (PTT) (L_n,T_) was determined as the ratio of the sum of the molar percentages of the soft and hard segments to the molar percentage of the soft segment [32]. The Equations are as follows.

*M*_h_ = (*I*_a_/2)/(*I*_c_/4) × 100%, (1)

*M*_s_ = 1 − *M_h_*, (2)

*W*_s_ = (*M*_s_ × 1130)/(*M*_s_ × 1130 + *M*_h_ × 206) × 100%, (3)

As can be seen from Table 1, with the increase in molecular weight of the PTMG segment, the w_s_ content of the soft PTMG segment in the polymer chains decreased from 54.8% to 45.7%, while the average sequence length of PTT-hard segments (L_n,T_) extended from 4 to 20. The degree of randomness (R) were calculated in the Supplementary Materials according to Equations (S1)–(S5) [33], and the R value is less than 1, as detailed in Table 1, indicating that the series of PTT-*b*-PTMG copolyesters is all block copolymers.

### 3.2. Melt and Crystallization Behavior PTT-b-PTMG Copolymers

**Figure 5:**
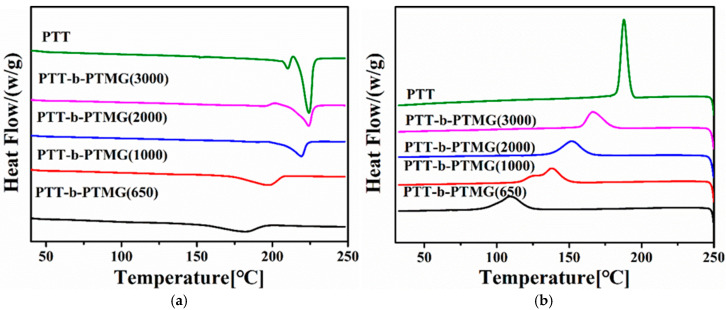
DSC thermograms of PTT-*b*-PTMG copolymers: (**a**) 2nd heating; (**b**) cooling.

As shown in Table 1, with the increase in the soft segment molecular weight will be followed with the increase in the average sequence length of the PTT rigid segment and the content of PTT-hard segments. The melt and crystallization behaviors of PTT-*b*-PTMG copolymers is dependent on the change in sequence structure of the rigid PTT segments. It is seen clearly that the melting and crystallization temperature (Table 2 and Figure 5) of PTT–b-PTMG copolymers shifted to a higher temperature with an increase in the flexible segment’s molecular weight. The enthalpy of melting and crystallization is slightly decreased with an increase in the flexible segment’s molecular weight.

**Figure 6:**
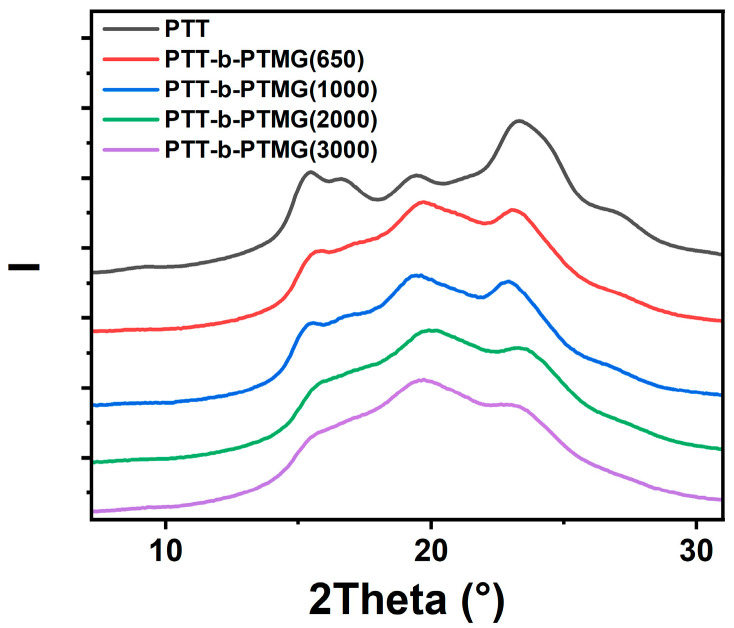
WAXS of PTT-*b*-PTMG copolymers with different PTMG molecular weights.

The crystal structures of PTT and PTT-*b*-PTMG copolymers prepared by injection molding were analyzed by WAXS, and the results are presented in Figure 6. The characteristic diffraction peaks of PTT appear at 2θ ≈ 15.6°, 16.7°, 19.6°, 21.8°, 23.6°, and 24.6°, which correspond to the (010), (0$\bar{1}$2), (012), (010), (010), (100), (102, 10$\bar{3}$), and (1$\bar{1}$3) lattice planes of PTT [34].

As the PTMG molecular weight increases, the diffraction peaks of the PTT-*b*-PTMG copolymers exhibit a slight reduction in intensity, indicating a gradual decrease in overall crystallinity. Importantly, no significant peak shifts are observed with increasing PTMG segment length, demonstrating that the fundamental lattice parameters of the PTT-hard-segment crystals remain unchanged, regardless of the molecular weight of the soft segment. Moreover, the principal reflections of PTT-*b*-PTMG(3000) are slightly sharper than those of the other samples. This indicates a reduction in lattice defects within the PTT-hard domains, which may result from more pronounced phase separation at the highest PTMG molecular weight.

**Figure 7:**
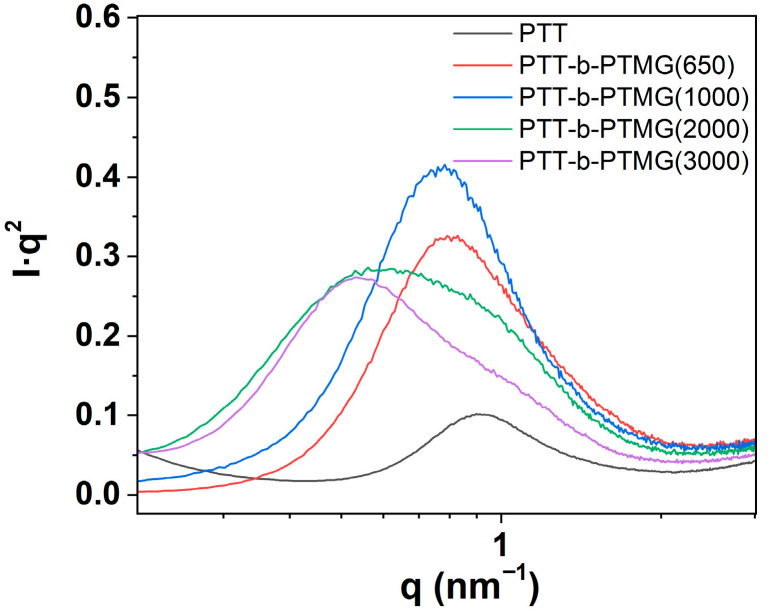
Lorentz-corrected SAXS profiles (I⋅q^2^ vs. q) of PTT and PTT-*b*-PTMG copolymers with different PTMG molecular weights.

The SAXS results (Figure 7) show that, as PTMG molecular weight increases, the position and lineshape of the scattering peak change noticeably, indicating changes in the long-period structure. Consistent with this, the long period *L* estimated from Bragg equation (L=2π/q) increases from ~7.5 nm (650 g/mol) to ~13.4 nm (3000 g/mol). For the higher-MW samples (2000–3000 g/mol), an additional low-q peak appears (~0.5 nm^−1^), while the shoulder at 0.80–0.95 nm^−1^ persists. This line-shape evolution suggests the coexistence of at least two characteristic spacings—a lamellar long period together with an additional, larger-scale spacing that may reflect hard—soft segregation beyond lamellar stacking.

These observations are consistent with DSC results. With increasing PTMG molecular weight, microphase separation appears stronger. This may promotes more uniform and better-developed crystallization within PTT-rich hard domains. As a result, the melting peak tends to narrow and the melting temperature shows a slight increase under our thermal protocol.

### 3.3. Phase Structure of PTT-b-PTMG Copolymers

**Figure 8:**
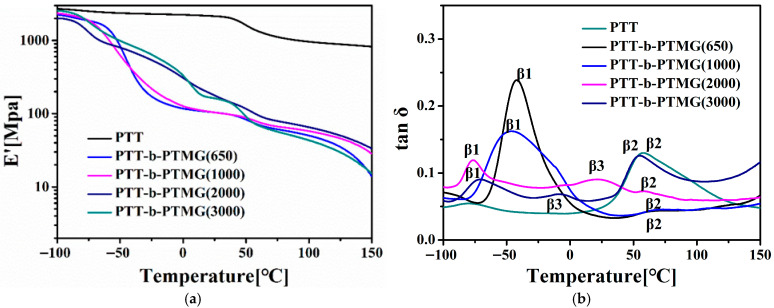
The storage modulus (**a**) and tan (**b**) as a function of temperature for PTT-*b*-PTMG copolymer.

Figure 8 illustrates the influence of PTMG’s soft-segment molecular weight on the dynamic mechanical behavior of the segmented PTT-*b*-PTMG copolymers, showing the storage modulus (E′) and loss tangent (tan δ) as functions of temperature. Over the entire temperature range examined, homopolymer PTT exhibited a single β-relaxation peak at approximately 58 °C, which corresponds to the glass transition temperature (Tg) of the PTT phase [26]. In the case of PTT-*b*-PTMG copolymers, the tan δ curves exhibit two or three—distinct β-relaxation transitions, labeled as β_1_, β_2_, and β_3_. These are assigned to: (i) the glass transition of the polyether-rich soft phase (β_1_), (ii) the glass transition of the amorphous polyester (PTT) domains (β_2_), and (iii) the melting of crystalline PTMG segments (β_3_). The β_3_ relaxation, observed in the temperature range of −25 to 25 °C, appears only in copolymers containing higher-molecular-weight PTMG segments and is attributed to the melting of PTMG crystallites—was confirmed by DSC in the Supplementary Materials (Figure S5) [34].

For PTT-*b*-PTMG copolymers containing PTMG segments with molecular weights of 650 and 1000 g/mol, the β_1_ relaxation peak appears at temperatures above the glass transition temperature of pure PTMG (−75 °C) [34]. In contrast, copolymers with PTMG segments of 2000 and 3000 g/mol exhibit β_1_ peaks close to −75 °C, aligning more closely with the Tg of neat PTMG. This shift is attributed to the presence of crystalline PTT-hard domains, which act as physical crosslinks that restrict the segmental mobility of the PTMG-soft phase, compounded by partial miscibility between the PTMG chains and the amorphous regions of the hard segments. Notably, in the copolymers with PTMG molecular weights of 2000 and 3000 g/mol, the β_1_ (at approximately −75 °C and −72 °C) and β_2_ (around 55 °C and 54 °C) relaxations are clearly resolved and remain largely unaffected by composition. This distinct separation of transitions indicates enhanced microphase segregation between the soft and hard domains, driven by the increased length—and thus reduced miscibility—of the higher-molecular-weight PTMG-soft segments. In order to further clarify their morphologies. The scanning electron microscopy (SEM) images of the copolymers was recorded. As can be seen in the Supplementary Materials (Figure S4), the microphase segregation of all of the copolymers were also observed. This result further confirms that the microphase separation strengthens with the increasing PTMG molecular weight, which is consistent with the SAXS and DMA results.

### 3.4. Thermal Stability of PTT-b-PTMG Copolymers

**Figure 9:**
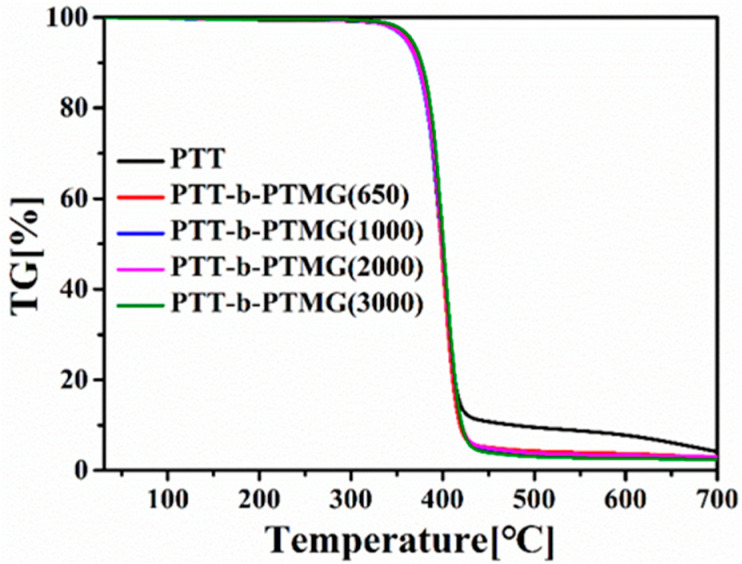
Mass loss (TG) curves of PTT-*b*-PTMG.

The thermal degradation behavior of the synthesized PTT-*b*-PTMG copolymers was investigated by thermogravimetric analysis (TGA) under an argon atmosphere (Figure 9). Key thermal parameters—including the temperatures at which 5%, 25%, and 50% mass loss occurred (denoted as T_5%_, T_25%_, and T_50%_), the char residue at high temperature (W_f_%), and the temperature of maximum degradation rate (T_max_)—are compiled in Table 3. The activation energy (Ea) for thermal degradation was calculated using the Freeman–Carroll method, as detailed in the Supplementary Materials (Equation (S6)) [35]. In an inert (argon) atmosphere, the calculated Ea values for the block copolymers are similar to that of the PTT homopolymer, indicating comparable thermal degradation mechanisms. As can be seen from Figure 9, the thermal degradation curve of the PTT-*b*-PTMG copolymer is similar to that of neat PTT. Within the temperature range of 700 °C, both exhibit one single weight loss step, corresponding to the degradation of the main chain. The T_5_% is considered the beginning of thermal degradation. The introduction of PTMG disrupts the regularity of the main chain, reduces the intermolecular forces between chains, and due to PTMG’s inherent poor thermal stability, collectively leads to a decrease in the initial thermal decomposition temperature of copolymers. Additionally, the incorporation of PTMG reduces the mass fraction of phenyl groups in the PTT-*b*-PTMG segment, resulting in a decline in its residual carbon rate. Regardless of composition, the T_50%_, T_max_, and char residue (W_f_%) values of the copolymers remain relatively consistent under an inert atmosphere. However, the initial degradation temperatures—T_5%_ and T_25%_—show an upward trend with increasing PTMG molecular weight, with the copolymer containing PTMG of 3000 g/mol exhibiting the highest values among the series. This improvement in early thermal stability is attributed to the longer average sequence length of the PTT-hard segments in the copolymer, which enhances the overall thermal resistance of the PTT-*b*-PTMG materials.

### 3.5. Mechanical Properties of PTT-b-PTMG Copolymers

**Figure 10:**
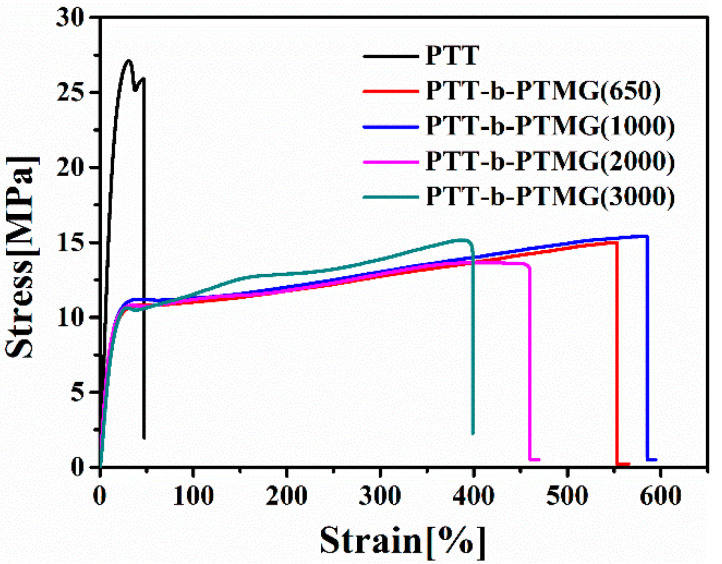
Tensile curves of PTT-*b*-PTMG with different PTMG molecular weights.

Representative stress–strain curves for the PTT-*b*-PTMG copolymer series are shown in Figure 10, with corresponding mechanical parameters—Young’s modulus, tensile strength, and elongation at break—summarized in Table 4. The shape of the stress–strain curves is clearly influenced by both the composition and the molecular weight of the PTMG-soft segment. As the molecular weight of PTMG increases within the copolymer backbone, Young’s modulus rises, while elongation at break generally decreases. Notably, tensile strength (stress at break) remains relatively unchanged for copolymers, regardless of PTMG molecular weight variation. Among the series, the copolymer with PTMG of 1000 g/mol exhibits the highest elongation at break, suggesting an optimal balance between soft and hard phases. In contrast, the sample with PTMG of 3000 g/mol displays a significantly higher Young’s modulus but markedly reduced ductility. This behavior is attributed to enhanced connectivity of the PTT-hard domains, resulting from the increased average sequence length of the hard segments, which restricts chain mobility and reduces extensibility.

**Figure 11:**
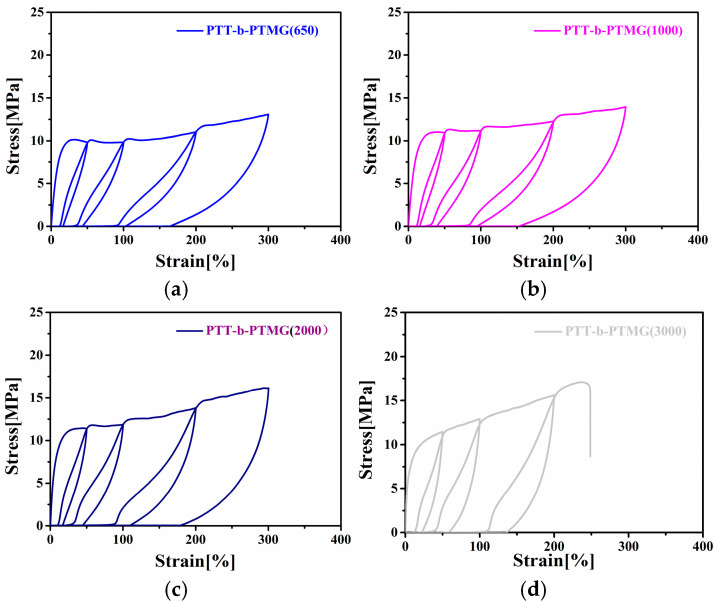
Cyclic tensile curves of PTT-*b*-PTMG with different PTMG molecular weights: (**a**) PTT-*b*-PTMG(650); (**b**) PTT-*b*-PTMG(1000); (**c**) PTT-*b*-PTMG(2000); (**d**) PTT-*b*-PTMG(3000).

**Figure 12:**
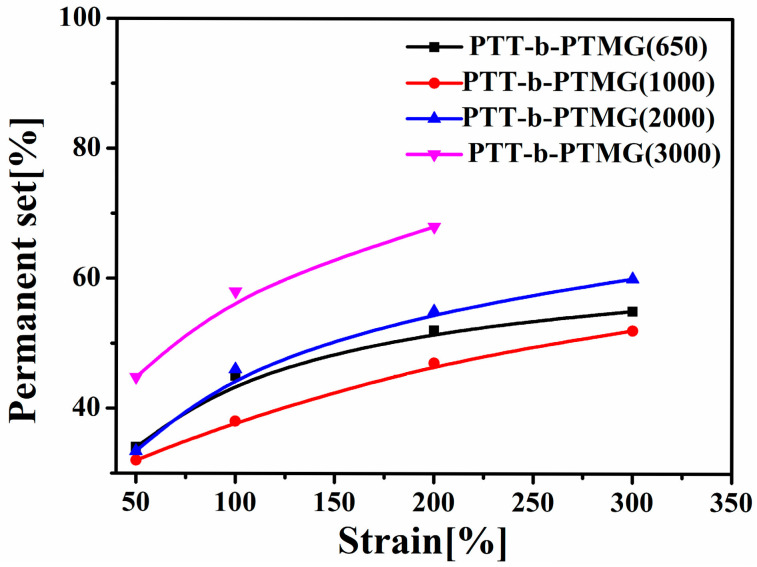
Tensile curves of PTT-*b*-PTMG.

The molecular weight of the PTMG-soft segment significantly affects the recovery behavior of PTT-*b*-PTMG block copolymers after large deformations (50%, 100%, 200%, and 300% strain), as illustrated by the cyclic tensile curves in Figure 11 and Figure 12. Among the series, copolymers incorporating PTMG segments with a medium molecular weight—specifically 1000 g/mol—demonstrate the best elastic recovery, evidenced by the lowest permanent set. In contrast, the copolymer containing PTMG of 3000 g/mol exhibits the highest permanent set, likely due to pronounced microphase separation, which hinders full retraction of the polymer chains upon unloading.

## 4. Conclusions

A series of PTT-*b*-PTMG-segmented block copolymers was successfully prepared via direct esterification followed by melt polycondensation, with the molecular weight of the PTMG-soft segment systematically varying from 650 to 3000 g/mol. This variation enabled the incorporation of 45.2–54.3 wt% PTMG into the copolymer backbone. Microphase-separated morphology was confirmed by dynamic mechanical analysis (DMA) and scanning electron microscopy (SEM), a structural feature essential for the materials’ elastomeric behavior and crystalline organization. Enhanced phase segregation between the soft amorphous PTMG domains and the rigid crystalline PTT segments was observed as the PTMG molecular weight increased. Concurrently, the average sequence length of the PTT-hard blocks also increased, leading to higher melting and crystallization temperatures in the copolymers. All synthesized copolyesters demonstrated excellent thermal stability and robust mechanical performance. Tensile testing further revealed that the extent of microphase separation strongly influences both the elastic modulus and shape recovery behavior. Importantly, the physical and mechanical properties of these materials can be precisely tailored by adjusting the molecular weight of the PTMG segment. The synthesized copolymers exhibit a favorable balance of properties, combining excellent elasticity with melt-processability, making them highly promising candidates for use as thermoplastic elastomers (TPEs).

## Figures and Tables

**Table 1:** Composition and physical properties of PTT-*b*-PTMG.

Sample	*W*_s_ (wt%)	*W*_h_ (wt%)	IV	*M*_n_ × 10^3^ (g/mol)	*M*_w_ × 10^3^ (g/mol)	*M*_w_/*M*_n_	*L_n,T_*	*R* ^C^
PTT	-	-	0.92	38.6	71.3	1.84	-	-
PTT-*b*-PTMG(650)	54.8	45.2	1.22	41.2	76.6	1.86	4	0.44
PTT-*b*-PTMG(1000)	49.6	50.4	1.27	43.9	81.3	1.85	6.5	0.43
PTT-*b*-PTMG(2000)	46.5	53.5	1.23	42.5	79.9	1.88	12.5	0.41
PTT-*b*-PTMG(3000)	45.7	54.3	1.25	43.6	82.8	1.90	20.0	0.36

*W*_s_—weight fraction of PTMG segments; *W*_h_—weight fraction of PTT-hard segments; *M*_n_—Number-Average Molecular Weight; *M*_w_—Weight-Average Molecular Weight; IV—intrinsic viscosity; *M*_w_/*M*_n_—polydispersity.

**Table 2:** Thermal performance parameters of PTT-*b*-PTMG.

Sample	*T*_m_/°C	Δ*H*_m_ (J·g^−1^)	*T*_p_/°C	Δ*H*_c_ (J·g^−1^)
PTT	224.0	43.5	187.8	−46.7
PTT-*b*-PTMG(650)	181.0	21.4	108.9	−28.5
PTT-*b*-PTMG(1000)	197.0	20.6	137.7	−29.1
PTT-*b*-PTMG(2000)	219.3	17.9	151.6	−26.6
PTT-*b*-PTMG(3000)	224.0	17.5	166.3	−23.6

*T*_p_—crystallizing temperature of copolymers; Δ*H*_m_—enthalpy of melting of copolymers; Δ*H*_c_—enthalpy of crystallization of copolymers; *T*_m_—melting temperature of copolymers.

**Table 3:** Thermal stability of PTT-*b*-PTMG.

Sample	T_5%_/°C	T_25%_/°C	T_50%_/°C	T_max_/°C	W_f_/%	Ea (R) (kJ/mol)
PTT	367.5	388.0	399.3	399.7	6.8	382 (0.996)
PTT-*b*-PTMG(650)	361.3	387.0	398.5	400.5	2.8	374 (0.997)
PTT-*b*-PTMG(1000)	360.2	386.6	399.6	400.3	2.5	380 (0.995)
PTT-*b*-PTMG(2000)	360.8	387.7	399.5	400.5	3.0	376 (0.996)
PTT-*b*-PTMG(3000)	366.0	389.8	400.6	401.2	2.5	390 (0.997)

T_5%_, T_25%_, T_50%_—temperature of the mass loss at 5%, 25% and 50%; T_max_%—the temperature of the maximum mass losses; W_f_%—char residue. Ea—energy activation; R—correlation coefficient in linear regression.

**Table 4:** Mechanical property parameters of PTT-*b*-PTMG.

Sample	E/MPa	σ/Mpa	ε/%
PTT	2012 ± 3.2	26 ± 0.3	49 ± 10
PTT-*b*-PTMG(650)	105 ± 3.5	15 ± 0.2	551 ± 16
PTT-*b*-PTMG(1000)	107 ± 2.6	14 ± 0.3	585 ± 14
PTT-*b*-PTMG(2000)	184 ± 2.1	15 ± 0.2	459 ± 14
PTT-*b*-PTMG(3000)	215 ± 2.3	15 ± 0.5	393 ± 12

E-Young’s modulus; σ-stress at break; ε-strain at break.

## Data Availability

The original contributions presented in this study are included in the article/Supplementary Material. Further inquiries can be directed to the corresponding author(s).

## Supplementary Materials

The following supporting information can be downloaded at: https://www.mdpi.com/article/10.3390/polym17202781/s1, Table S1: The polymerization parameters of of PTT-*b*-PTMG copolymers; Figure S1: Chemical structural formula of PTT-*b*-PTMG; Figure S2: ^13^C-NMR spectra of PTT-*b*-PTMG; Figure S3: ^13^C-NMR spectrum of PTT-*b*-PTMG; Figure S4: The SEM images of PTT-*b*-PTMG copolymers; Figure S5: DSC thermograms of PTT-*b*-PTMG copolymers of 2nd heating.
