# Supplementary material for: The Effects of Soft-Segment Molecular Weight on the Structure and Properties of Poly(trimethylene terephthalate)-block-poly(tetramethylene glycol) Copolymers
Source: Polymers (Basel). 2025 Oct 17;17(20):2781. doi: 10.3390/polym17202781 (PMC12567339; doi:10.3390/polym17202781)
Supplement: Supplementary file 1 [file polymers-17-02781-s001.zip › polymers-3890344-supplementary.pdf]

Supplementary Materials

The Effects of Soft- Segment Molecular Weight on the Structure and Properties of Poly(trimethylene terephthalate)-block-poly(tetramethylene glycol) Copolymers

Hailiang Dong [1,2], Yuchuang Tian [2], Junyu Li [2], Jiyou Shi [2], Jun Kuang [2,*], Wenle Zhou [2] and Ye Chen [1,*]

1. State Key Laboratory of Advanced Fiber Materials, College of Materials Science and Engineering, Donghua University, Shanghai 201620, China; lddonghailiang@163.com
2. Sinopec Shanghai Research Institute of Petrochemical Technology Co., Ltd., Shanghai 201208, China; tianych.sshy@sinopec.com (Y.-c.T.); lijy.sshy@sinopec.com (J.-y.L.); shijy.sshy@sinopec.com (J.S.); zhouwl.sshy@sinopec.com (W.-l.Z.)

* Correspondence: kuangj.sshy@sinopec.com (J.K.); chenye@dhu.edu.cn (Y.C.); Tel.: +86-186-1693-6252 (J.K.); +86-139-1778-9096 (Y.C.)

Table S1. The polymerization parameters of of PTT-b-PTMG copolymers

Sample	Esterification Yields（%）	polymerization yields（%）
PTT	95±1.3	75±5.0
PTT-b-PTMG (650)	94±1.2	74±6.5
PTT-b-PTMG (1000)	96±1.4	73±7.0
PTT-b-PTMG (2000)	95±1.5	72±7.5
PTT-b-PTMG (3000)	96±1.6	72±8.2

* T_p–crystallizing temperature of copolymers; ΔH_m–enthalpy of melting

The molar fraction f can be determined from the integrated areas of the corresponding resonance signals in the 13C NMR spectrum. Specifically, the molar fraction of the PDO–PTA units, denoted as f(PT), and that of the PTMG–PTA units, denoted as f(G-T), are calculated using the following equations, respectively[1]:

$$f\,(\text{PT}) = f\,(\text{P-T-P}) + 0.5 f\,(\text{P-T-G}) \tag{S1}$$

$$f\,(\text{GT}) = f\,(\text{G-T-G}) + 0.5 f\,(\text{P-T-G}) \tag{S2}$$

The degree of randomness (R) of the PTT-b-PTMG copolyesters can be expressed by the following equation:

$$R = \frac{f\,(\text{P-T-G})}{2f\,(\text{P-T})} + \frac{f\,(\text{P-T-G})}{2f\,(\text{G-P})} \tag{S3}$$

The average sequence length of hard segment PTT units and soft segment PO3G units in a molecular chain can be calculated by the following equation:

$$L_{PT} = 1 + \frac{2f\,(\text{P-T})}{f\,(\text{P-T-G})} \tag{S4}$$

$$L_{GT} = 1 + \frac{2f\,(\text{G-T})}{f\,(\text{P-T-G})} \tag{S5}$$

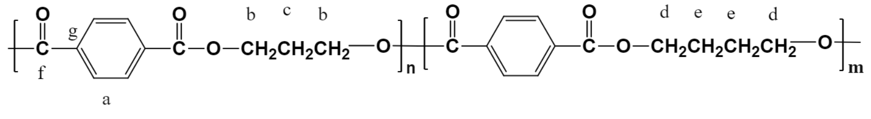

Figure S1. Chemical structural formula of PTT-b-PTMG

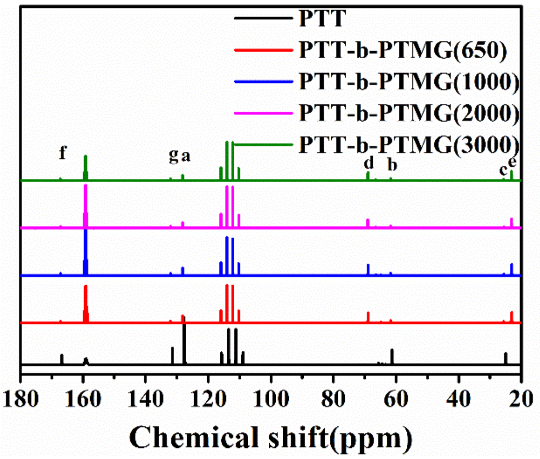

Figure S2. ^{13}C-NMR spectra of PTT-b-PTMG

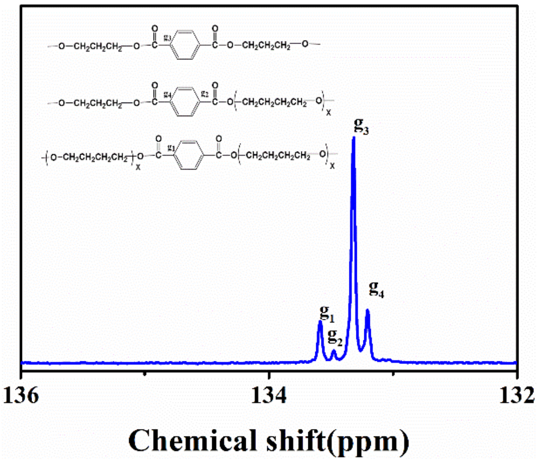

Figure S3. ^{13}C-NMR spectrum of PTT-b-PTMG

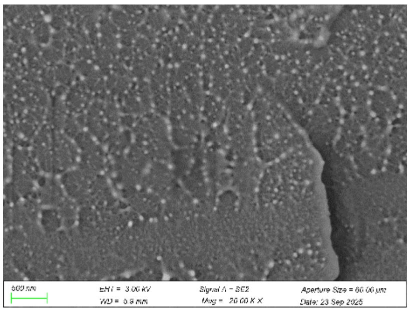

(**a**)

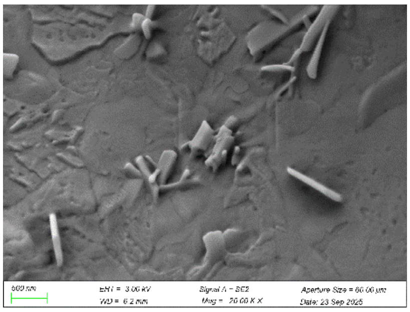

(**b**)

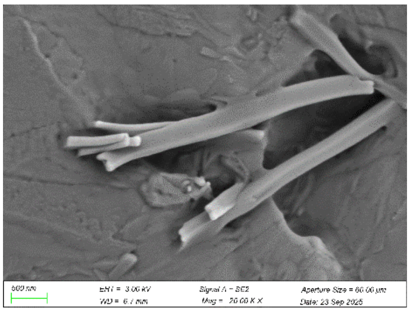

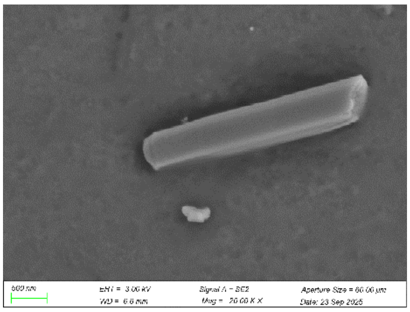

(c) (d)

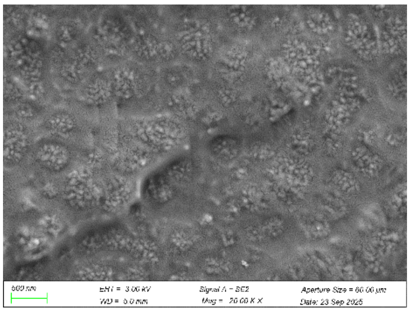

(e)

Figure S4. The SEM images of PTT-b-PTMG copolymers: (a) PTT; (b) PTT-b-PTMG(650); (c) PTT-b-PTMG(1000); (d) PTT-b-PTMG(2000); (e) PTT-b-PTMG(3000).

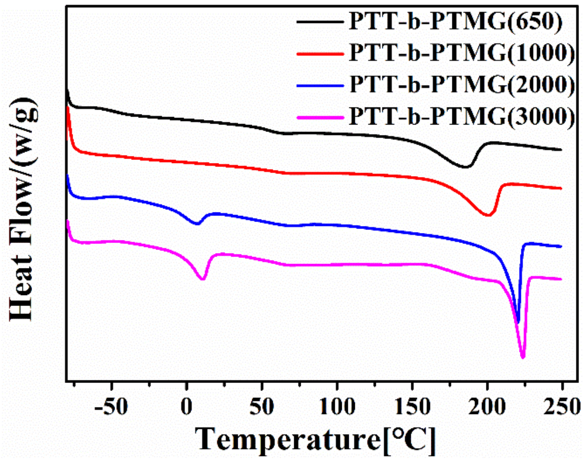

Figure S5. DSC thermograms of PTT-b-PTMG copolymers of 2nd heating.

The Ea was calculated according Freeman–Carroll method with the following equation[2]:

$$\frac{\Delta logdw/dt}{\Delta \log w_r} = \text{n- } Ea/2.303 \frac{\Delta \ (\frac{1}{T})}{\Delta \log w_r} \tag{S6}$$

where, dw/dt represents the rate of weight loss with respect to time. The term w_r is defined as w_c–w, where wc is the total weight loss at the end of the decomposition (or at a specified reaction time), and w is the cumulative weight loss at time t.The parameter n denotes the reaction order, while T and R correspond to the absolute temperature and the universal gas constant, respectively. According to the Freeman–Carroll method, a plot of [Δlog(dw/dt)/Δlogw_r] against [Δ(1/T)/Δlogw_r] yields a straight

line. The slope of this line is directly used to determine the activation energy (Ea) via the relationship: Ea=−slope×2.303×R.

References

1. Li, Y.; Liu, Y.; Liu, A.; Xu, C.; Zhang, C.; Yu, J.; Yuan, R.; Li, F. Poly(trimethylene terephthalate-b-poly(trimethylene ether) glycol) copolymers: From bio-based thermoplastic elastomers to elastic fibers for apparel. *European Polymer Journal* **2025**, *225*, doi:10.1016/j.eurpolymj.2024.113706.
2. Paszkiewicz, S.; Szymczyk, A.; Irska, I.; Pawlikowska, D.; Piesowicz, E. Synthesis, structure, and physical properties of poly(trimethylene terephthalate)-block-poly(caprolactone) copolymers. *Journal of Applied Polymer Science* **2018**, *136*, doi:10.1002/app.47341.
